# Study on the cross-linking process of carboxylated polyaldehyde sucrose as an anti-wrinkle finishing agent for cotton fabric

**DOI:** 10.1038/s41598-022-09216-7

**Published:** 2022-03-30

**Authors:** Jiangfei Lou, Dan Wang, Xuerong Fan

**Affiliations:** Key Laboratory of Science and Technology of Eco-Textiles, Ministry of Education, Jiangnan University, Wuxi, 214122 Jiangsu China

**Keywords:** Chemistry, Process chemistry, Chemical synthesis

## Abstract

Sucrose was oxidized in a two-step oxidation reaction catalyzed by 2,2,6,6-tetramethyl-1-piperidinyloxy (TEMPO)-laccase and sodium periodate (NaIO_4_). To generate carboxylated polyaldehyde sucrose (openSu) containing multiple aldehyde and carboxyl groups. The amount of TEMPO and laccase used, as well as the temperature and reaction time were optimized for the oxidation reaction. The successful combination of aldehyde and carboxyl groups of openSu with cellulose was achieved by changing the composition, ratio of the catalyst and the curing conditions. Thereafter, we analyzed the structural characteristics of openSu as well as the aldehyde and carboxyl group content using nuclear magnetic resonance carbon spectroscopy (^13^C NMR). We found that the optimal finishing conditions were a mixture of magnesium chloride and sodium hypophosphite at a mass concentration ratio of 16 g/L:4 g/L, and curing at 150 °C for 3 min followed by curing at 180 °C for 2 min. There was significant improvement in the anti-wrinkle performance of the openSu-finished fabric, with a wrinkle recovery angle of 258°, whiteness index of 72.1, and a tensile strength rate of more than 65%. We also studied the covalent crosslinking mechanism between openSu and the cotton fabrics.

## Introduction

High-quality development is the trend of modern textiles, and it is also an effective way and the urgent need to improve the product grade and added value of the textile industry and reform the supply-side structure. This is due to improved living standards and high-paced working environments that demand high quality fabrics and clothing that are easy to take care of and have superior shape retention capacity^1,2^. Cotton fabrics are examples of textile fibers that are popular for their excellent properties such as softness, comfort, breathability, and good moisture absorption^3,4^. However, cotton products have poor resilience, large shrinkage, and are prone to wrinkling thus requiring frequent ironing. These cause a lot of inconveniences in the modern high-paced environment. Therefore, shape retention is a key attribute of high-quality cotton fabrics. The shape retention capacity of cotton fabrics can be improved by subjecting the fabrics to non-iron finishing processes^5–7^, an area of active research in the finishing of fabrics.

At present, etherified dimethyl dihydroxy ethylene urea resin (DMDHEU) is commonly used as an anti-wrinkle finishing agent. However, the use of DMDHEU during the finishing process releases formaldehyde, which is an environmental pollutant and harmful to human health^8–10^. As a result, other anti-wrinkle agents such as polycarboxylic acid, amino silicone oil and epoxy resin have been developed. Unfortunately, the finishing effects of the new agents are not as good as DMDHEU, and they are associated with significant loss in fabric strength^1,3,5^. Among the formaldehyde-free anti-wrinkle finishing agents, polycarboxylic acids have the greatest potential to replace urea–formaldehyde resins. This is because there has been a lot of research on polycarboxylic acids and widespread applications leading to their fast development. BTCA is a polycarboxylic acid that has attracted the most attention. However, high production costs, the pressure on the environment caused by the use of phosphorus-containing catalysts, and the significant loss of fabric strength associated with BTCA has limited its industrial applications^1,11^.

Sucrose is a disaccharide that is formed through the dehydration of one molecule of glucose and one molecule of fructose. According to previous studies, the primary hydroxyl group of cellulose can be selectively oxidized by the TEMPO-laccase system to obtain carboxyl cellulose^12,13^, while the adjacent hydroxyl group of the glucose ring can be selectively oxidized by sodium periodate to form dialdehyde derivatives^14–18^. Therefore, we designed cross-linking agents based on the molecular structure and cross-linking mechanisms of polyaldehydes and polycarboxylic acids. The aim was to generate a new formaldehyde-free finishing agent that can be used to develop new anti-wrinkle methods and reaction systems.

In this paper, we analyzed the selective oxidation of carboxylated polyaldehyde sucrose (openSu) using TEMPO-NaIO_4_. The primary hydroxyl group of sucrose was first oxidized using the TEMPO-laccase system to form 6,6′-carboxy sucrose (oxySu). NaIO_4_ was then used to oxidize oxySu to produce openSu. The carboxyl and aldehyde composition of openSu was determined through potentiometric titration with NaOH, while the structural characteristics of openSu were analyzed using ^13^C NMR. We also evaluated the effects of different ratios of the components of the catalytic system, and curing conditions (temperature and time) on the wrinkle recovery angle (WRA), whiteness index (WI) and tensile strength (TS) of the fabrics. The effects of DMDHEU, glutaraldehyde (GA) and BTCA on the WRA, WI, TS and hydrophilicity (wetting time) of treated fabrics were also compared under different curing conditions.

## Results and discussion

### Effect of reaction conditions on the carboxyl groups content

**Figure 1:**
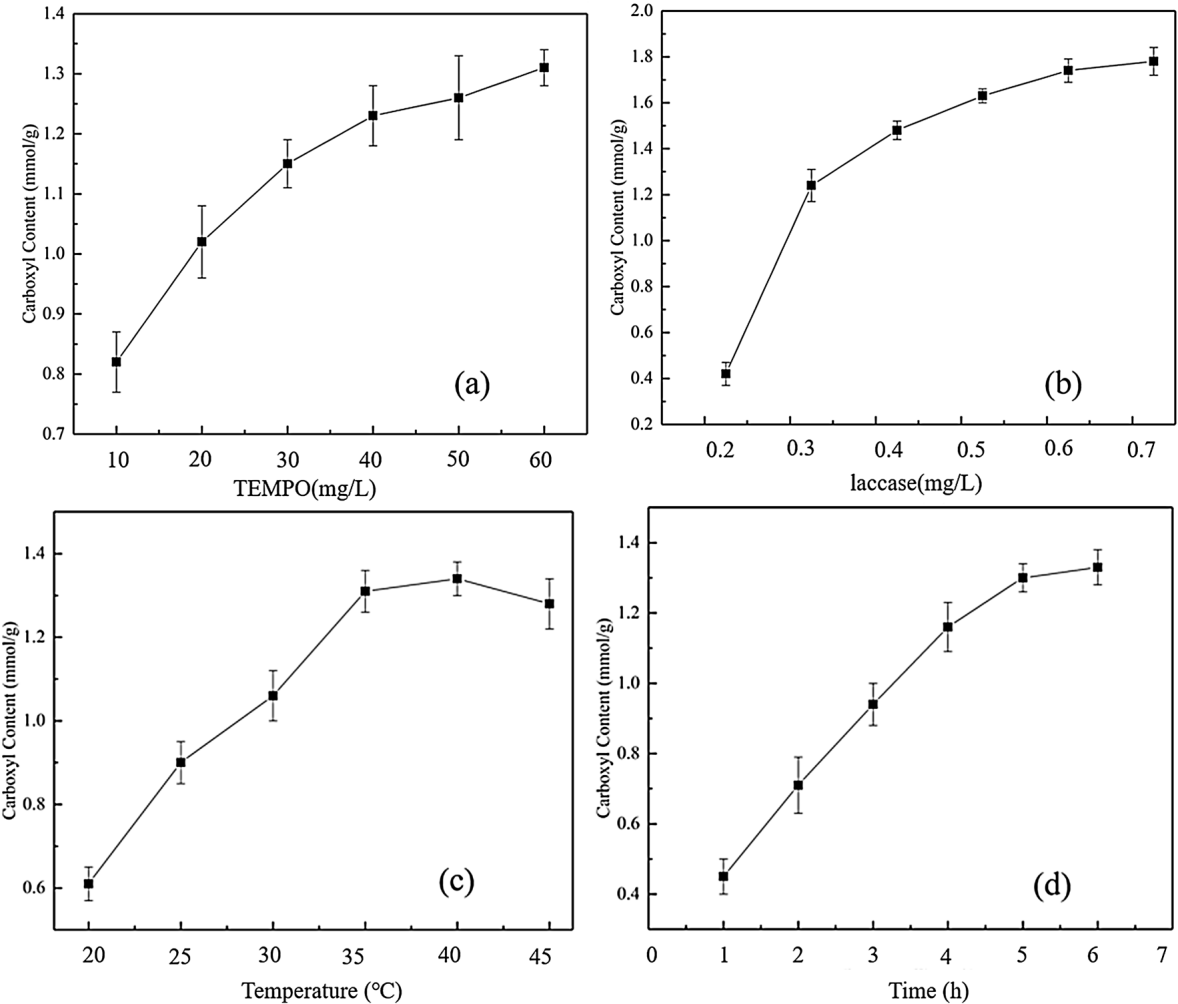
Effect of TEMPO factor (**a**), laccase factor (**b**), reaction temperature (**c**) and reaction time (**d**) on the carboxyl content of openSu.

The primary hydroxyl groups of sucrose were first oxidized to aldehyde groups using the TEMPO system, and then further converted to carboxyl groups^13,14,17^. To explore changes in carboxyl group content in the TEMPO oxidation process, a single-factor optimization experiment was carried out. The reaction conditions are shown in Table 1. The changes in the carboxyl content of oxidized sucrose under different conditions are presented in Fig. 1.

**Table 1:** Oxidizing conditions of the TEMPO system.

Factors	Reaction conditions
TEMPO (mg/L)	10, 20, 30, 40, 50, 60
Laccase (mg/L)	0.2, 0.3, 0.4, 0.5, 0.6, 0.7
Reaction temperature (°C)	20, 25, 30, 35, 40, 45
Reaction time (h)	1, 2, 3, 4, 5, 6

Figure 1a shows the relationship between the concentration of TEMPO and the carboxyl group content of openSu. There was a significant increase in the number of carboxyl groups in openSu with increase in the amount of TEMPO. The carboxyl group content of openSu reached 1.2 mmol/g when the amount of TEMPO was 8 mg. This was because in the TEMPO oxidation system, the reduced TEMPO reacted with NaClO to generate nitrogen carbonyl cations (TEMPO^+^), which then further reacted with the primary hydroxyl groups of the sucrose^16,19^. According to the “effective collision” theory, increasing the amount of TEMPO increased the number of activated molecules per unit volume which in turn increased the effective number of collisions between tempo and sucrose per unit time. Consequently, more nitrogen carbonyl cations were produced leading to accelerated carboxyl formation during the oxidation process.

The carboxyl content of openSu was also affected by the amount of laccase as shown in Fig. 1b. There was a rapid increase in the carboxyl group content of openSu as the amount of laccase increased from 0.04 to 0.10 mg. This was because laccase had an important role in the formation of TEMPO^+^^13,14^. An increase in the amount of laccase accelerated the oxidation reaction, since more aldehyde groups were generated in the same reaction time. The generated aldehyde groups were further oxidized to carboxyl groups, thereby increasing the carboxyl group content of openSu. From this experiment, we found that the maximum carboxyl group content of 1.3 mmol/g was attained when the amount of laccase ranged from 0.10 to 0.12 mg.

The effect of the reaction temperature on the carboxyl content of openSu was ill in Fig. 1c. As the reaction temperature increased, the carboxyl content was also increased rapidly. This was because biological enzymes have their optimum reaction temperature, and the optimum temperature of laccase was 30–35 °C. At this temperature range, the carboxyl group content can reach the maximum value of 1.7 mmol/g^14^.

Figure 1d shows the effect of reaction time on the carboxyl content of openSu. The figure shows that there was an initial increase in the carboxyl group content as the reaction time increased but after a certain time point the content leveled off. During the first 60 min of the oxidation process, the carboxyl content of openSu increased rapidly and reached the maximum value (1.18 mmol/g). However, there was no significant change in the carboxyl content when the reaction time was extended to 90 min. This was because the number of directly accessible groups decreased, and increased steric hindrance prevented the combination of TEMPO^+^ with the primary hydroxyl groups, resulting in decreased oxidation rate.

### Aldehyde groups content of openSu

**Figure 2:**
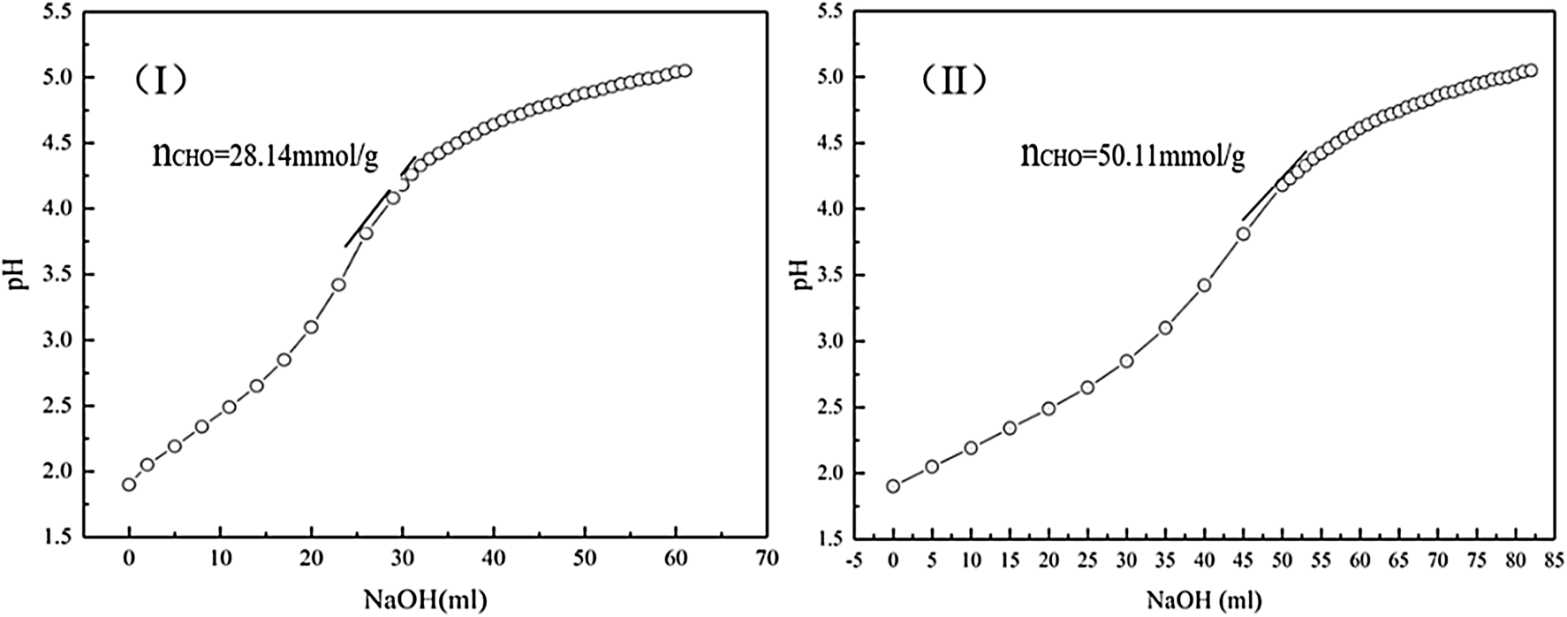
Aldehyde content of OSu (**I**) and openSu (**II**).

NaIO_4_ is an inorganic salt containing multiple aldehyde groups. In our previous study^20,21^, we successfully used NaIO4 to selectively oxidize dialdehyde sucrose (OSu). However, the aldehyde groups that were formed self-polymerized during the reaction process, thus reducing the final aldehyde group content of OSu. Therefore, we changed the molecular structure of OSu, by introducing the carboxyl groups to decrease the reaction between the aldehyde and hydroxyl groups, in this study. Figure 2 shows the aldehyde contents of openSu and OSu.

Form the Fig. 2, we see that the aldehyde content of openSu was 50.11 mmol/g according to Eq. (2), while the aldehyde content of OSu was only 28.14 mmol/g (Fig. 2). These finding indicated that the introduction of carboxyl groups through the TEMPO-laccase system reduced the polymerization of the aldehyde groups during the oxidation of NaIO4, and greatly increased the aldehyde group content of openSu.

### Structural characteristics of openSu

**Figure 3:**
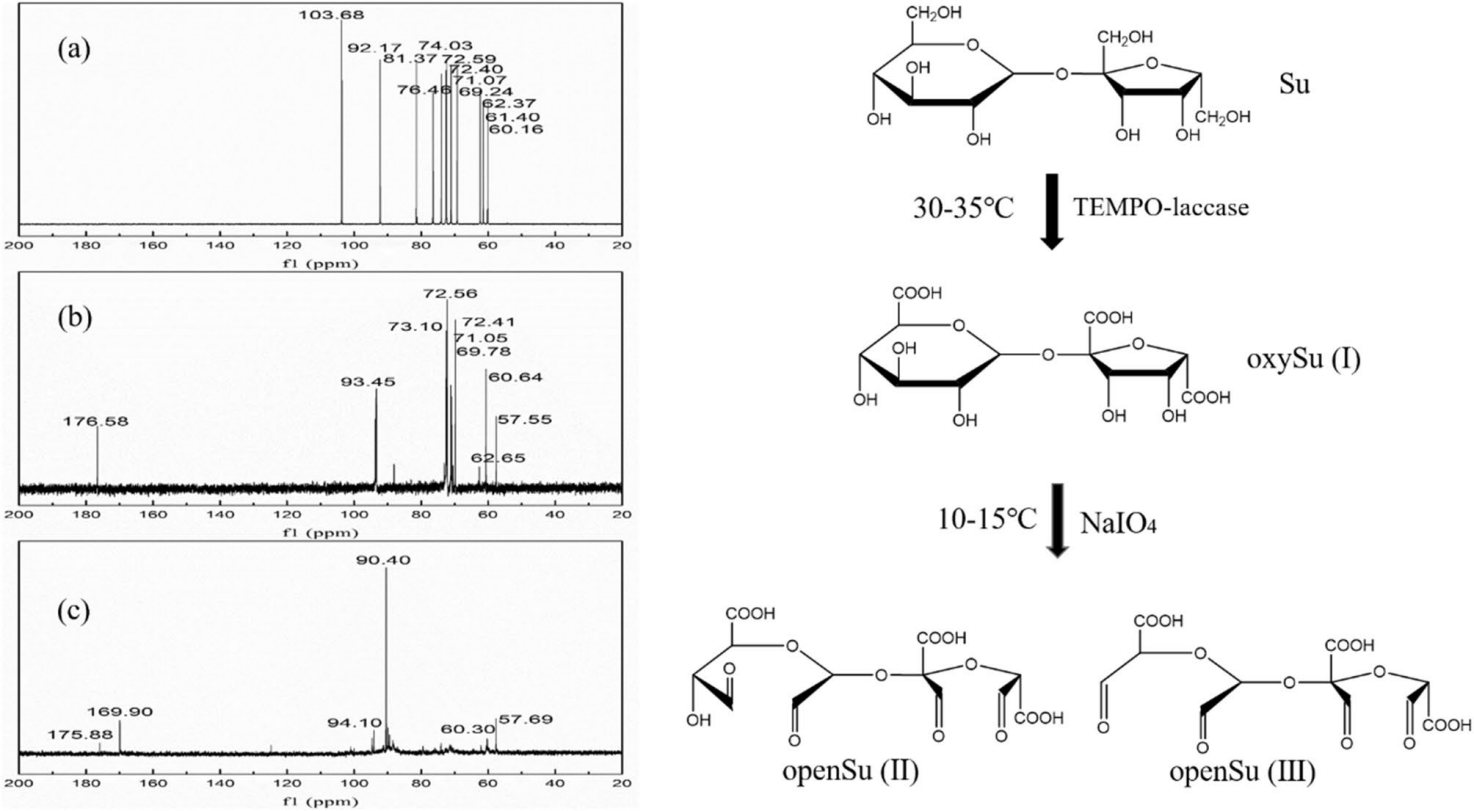
^13^C NMR spectra of sucrose (**a**), oxySu (**b**) and openSu (**c**) and the oxidation process of sucrose.

In the ^13^C NMR spectrum, carboxyl and aldehyde groups have characteristic chemical shift peak patterns. We compared the carboxyl and aldehyde group contents among sucrose, oxySu and openSu using ^13^C NMR spectrum analysis (Fig. 3), the oxidation mechanism was also shown in Fig. 3.

From Fig. 3a,b, the characteristic chemical shift at 177.60 ppm was attributed to the carboxyl group (–COONa), which was selectively oxidized by the TEMPO-laccase system^13,14,22^. At 57.60 ppm, the new peak was assigned to the CH_2_–O–C group, indicating that the C-6 aldehyde group had reacted with the primary hydroxyl group during the oxidation process. A comparison between Fig. 3b,c showed a characteristic chemical shift at 169.81 ppm which was attributed to the aldehyde group (–CHO) of openSu^14,23^. This proved that oxySu had been successfully oxidized using the NaIO_4_ selective oxidation system, and that the expected carboxylated polyaldehyde sucrose had been obtained.

### Effect of the mass concentration ratio of MgCl_2_ and SHP

**Figure 4:**
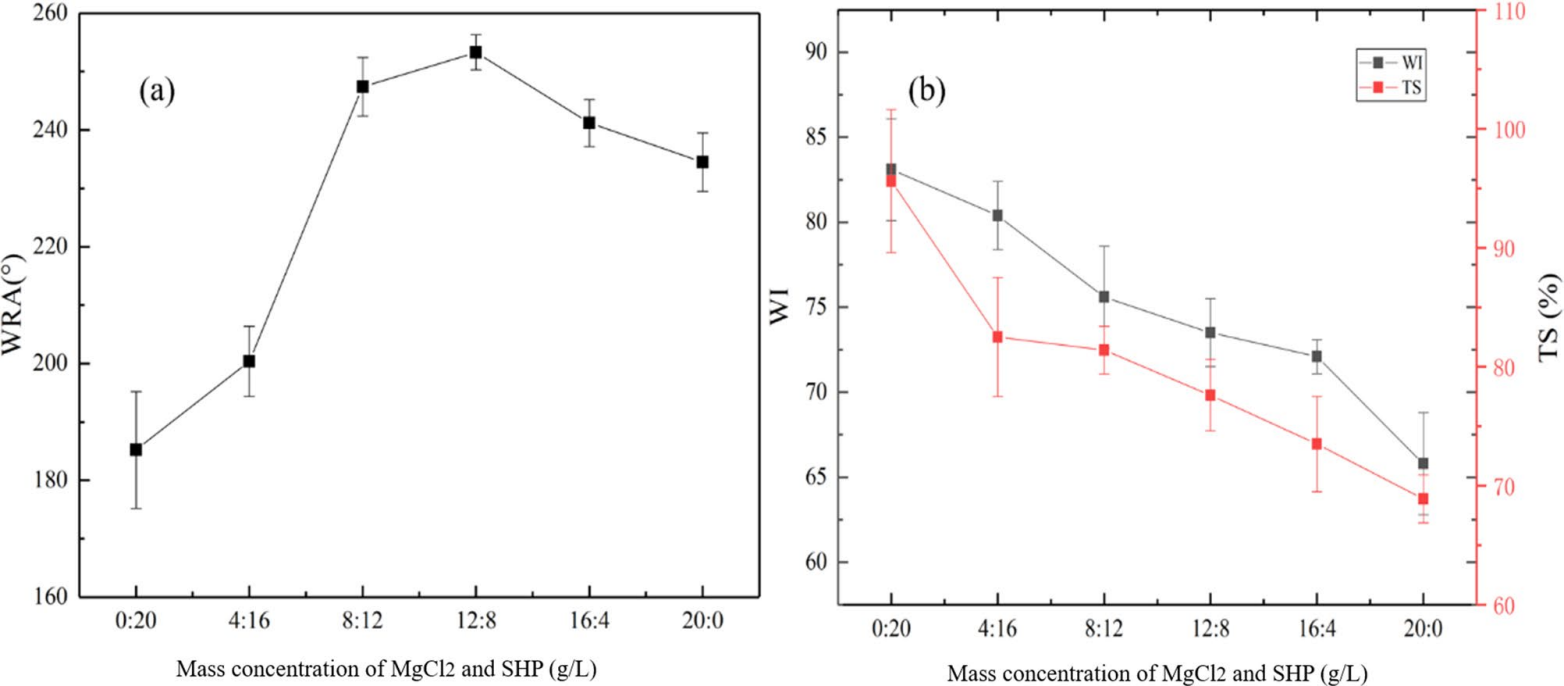
The effects of different mass concentration ratios for MgCl_2_ and SHP, on the WRA (**a**), WI and TS (**b**) of the finished fabrics.

Since openSu contains carboxyl and aldehyde groups, our aim was to combine all the groups with cellulose to improve the anti-wrinkle performance of the openSu-finished fabric. According to literature^1,3,5,20^, SHP catalyze the combination of carboxyl and hydroxyl groups, while MgCl_2_ catalyzes the combination of aldehyde and hydroxyl groups. Therefore, to catalyze the combination of openSu and cellulose, a mixture of SHP and MgCl_2_ was selected. We then evaluated the anti-wrinkle property of the finished fabric when different mass concentration ratios of MgCl_2_ and SHP were used (the curing conditions was 3 min at 160 °C) (Fig. 4).

There was only a slight increase in the WRA (183° of openSu-finished fabric compared to 128 ± 5° of the control fabrics) of the finished fabric when the mass concentration ratio of MgCl_2_ to SHP was 0:20 g/L (Fig. 4a). This was because the aldehyde groups of openSu could not react with the hydroxyl groups of cellulose in the absence of MgCl_2_. A mass concentration ratio of 12 g/L:8 g/L significantly increased the WRA of the finished fabric to the maximum value of 253°. Under this condition, the aldehyde and carboxyl groups of openSu were able to fully combined with cellulose, and improve the wrinkle property of the finished fabric. On the other hand, a mass ratio of 16 g/L:4 g/L reduced the WRA of the fabric since there was a lack of sufficient SHP to catalyze the combination of the carboxyl groups of openSu with cellulose. There was also a significant reduction in WRA of the fabric when the mass concentration ratio was 20 g/L:0, since the carboxyl groups of openSu could not combined with cellulose.

Figure 4b shows the changes in WI and TS of the finished fabric as the ratio of MgCl_2_ to SHP changed. An increase in MgCl_2_ decreased the WI and TS of the fabric. This was because the presence of MgCl_2_ accelerated the combination of aldehyde groups and cellulose which increased the reaction rate of the hydroxyl groups in the cellulose. In addition, the cellulose underwent dehydration and condensation to produce some colored substances, which reduced the WI of the openSu-treated fabric. During the finishing process of openSu and cellulose, the pH of the finishing solution was low (pH was 3–4), and the cloth surface pH of the finished fabric was also low. This resulted in non-uniform hydrolysis of cellulose during the curing process, which significantly reduced the TS.

### Effect of the curing temperature and time

Curing temperatures of 180 °C and 150 °C are required in the anti-wrinkle finishing of polycarboxylic acids and dialdehydes, respectively, while the curing time ranges from 1 to 3 min^1,5,11^. Since openSu fabrics have polycarboxylic acids and dialdehydes, curing at 150 °C and 180 °C was required. Therefore, combination of the aldehyde and carboxyl groups of openSu with cellulose happened at 150 °C and 180 °C, respectively. This generated openSu that was fully combined with cellulose. The anti-wrinkle properties of openSu-finished fabrics under different curing conditions are shown in Table 2.

**Table 2:** Anti-wrinkle properties of treated cotton fabrics under different curing conditions.

Finishing agent (g/L)	Catalysis (g/L)	Curing condition	Curing condition	WRA (warp + weft) (°)	WI	TS (%)	
						warp	weft
openSu = 80	SHP: MgCl_2_ = 4:16	1 min at 150 °C	1 min at 180 °C	226 ± 3	74.5 ± 0.3	72.3 ± 0.1	68.1 ± 0.4
		1 min at 150 °C	2 min at 180 °C	235 ± 4	72.1 ± 0.2	67.5 ± 0.3	65.3 ± 0.5
		1 min at 150 °C	3 min at 180 °C	241 ± 5	70.1 ± 0.4	64.6 ± 0.4	62.5 ± 0.3
		2 min at 150 °C	1 min at 180 °C	249 ± 3	74.5 ± 0.3	70.1 ± 0.2	66.4 ± 0.2
		2 min at 150 °C	2 min at 180 °C	253 ± 2	72.3 ± 0.5	68.2 ± 0.4	64.1 ± 0.3
		2 min at 150 °C	3 min at 180 °C	258 ± 6	70.8 ± 0.6	63.4 ± 0.3	61.3 ± 0.5
		3 min at 150 °C	1 min at 180 °C	242 ± 3	69.5 ± 0.4	67.2 ± 0.2	63.6 ± 0.3
		3 min at 150 °C	2 min at 180 °C	258 ± 2	67.4 ± 0.5	65.3 ± 0.5	58.9 ± 0.6
		3 min at 150 °C	3 min at 180 °C	260 ± 4	55.4 ± 0.3	55.1 ± 0.3	51.3 ± 0.4
		3 min at 150 °C	–	234 ± 5	72.6 ± 0.2	68.4 ± 0.5	63.2 ± 0.3
		–	2 min at 180 °C	183 ± 3	82.6 ± 0.3	81.5 ± 0.4	75.7 ± 0.5
		–	–	128 ± 4	87.5 ± 0.5	100.0	100.0

There was an increase in the WRA but a decrease in the WT and TS of the finished fabric when the curing time was increased at a constant temperature of 150 °C (Table 2). This was because the longer curing time allowed openSu to fully combined with cellulose, while the increased hydrolysis of the fabric at high temperatures decreased TS. After curing at 150 °C for 3 min, the WRA of the fabric was higher. The finished fabric has a similar pattern when it is cured at 180 °C. When it was cured at 180 °C for 2 min, the WRA of the fabric was higher than the un-curied fabric. After openSu finishing, the fabric was cured separately at 150 °C for 3 min and 180 °C for 2 min, the WRA of the fabric was improved. These findings indicate that the MgCl_2_ and SHP-catalyzed combination of the aldehyde and carboxyl groups of openSu with cellulose improved the anti-wrinkle properties of the openSu-finished fabric.

Calculated the *P* value of WRA statistical data of unfinished fabric, 150 °C finishing and 180 °C finishing fabric respectively, P_1_ was the *P* value of WRA of unfinished fabric and 150 °C finishing, P_2_ was the *P* value of WRA of unfinished fabric and 180 °C finishing, P_3_ was the *P* value of WRA of 150 °C finishing and 180 °C finishing, and P_1_ was 0.00003363, P_2_ was 0.000246, P_3_ was 0.006497. It was shown that the correlation between openSu finishing fabric and curing at 150 °C was higher than the correlation with curing at 180 °C, which was also related to the high content of aldehyde groups in openSu, which was the main crosslinking group. At the same time, P_3_ was more than 0.005, indicating that the correlation between 150 and 180 °C was not obvious. Therefore, the curing time at 150 °C should be longer than 180 °C.

Combining the WRA, WI and TS of openSu-finished fabrics with different curing temperatures and time in Table 2, we have concluded that the optimal curing method was two-steps curing methods, that was, curing at 150 °C for 3 min and curing at 180 °C for 2 min.

### The effect of different finishing agents on the anti-wrinkle performance of openSu

**Figure 5:**
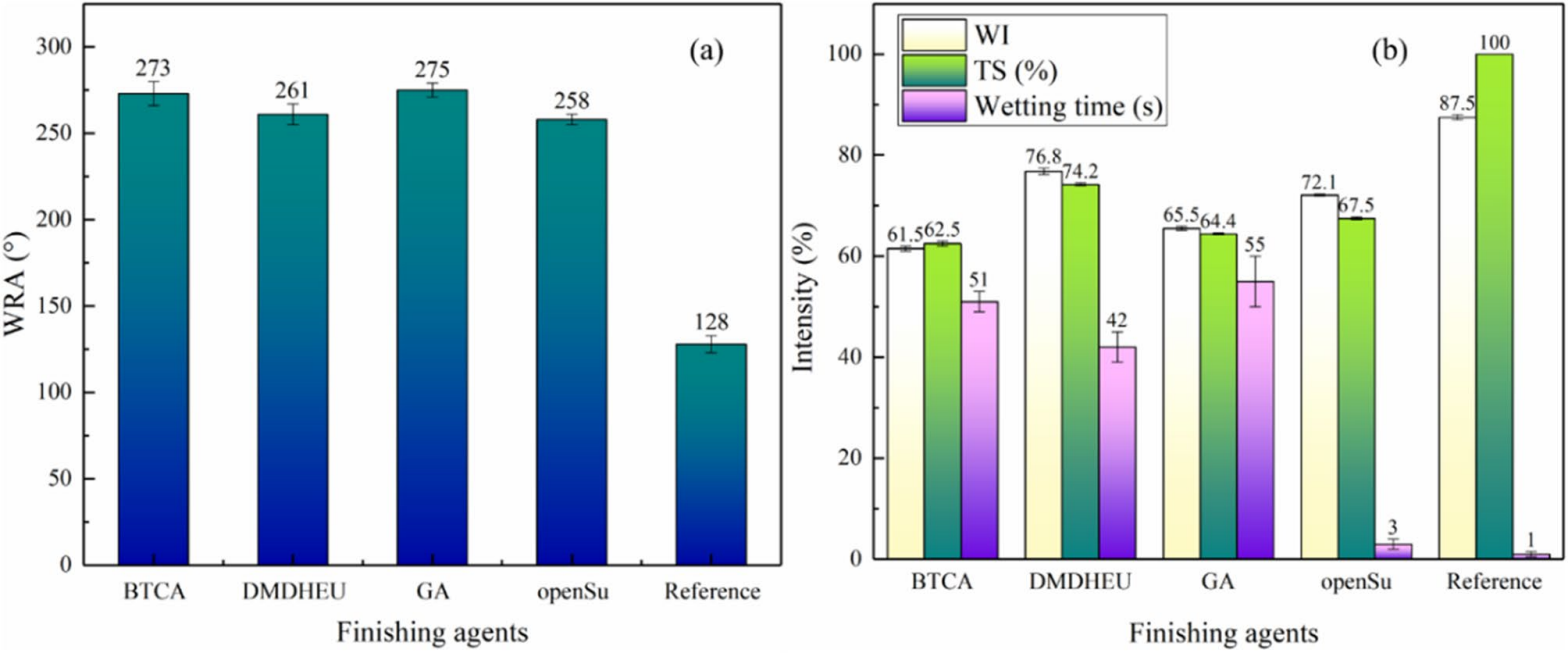
The WRA (**a**), WI, TS and wetting time (**b**) of the finished fabrics.

We further analyzed the effects of BTCA, DMDHEU and GA on the anti-wrinkle properties of openSu-treated fabrics by comparing the WRA, WI, TS and wetting time of the finished fabrics. The anti-wrinkle finishing solution contained BTCA, GA or DMDHEU and the corresponding catalyst (MgCl_2_ or SHP) in addition to 80 g/L openSu. The reference fabric was finished with water. The WRA, WI and TS of the finished samples were measured, and are shown in Fig. 5.

The WRA of openSu-treated fabric cured at 150 °C and 180 °C was 258°, which was almost similar to the WRA of DMDHEU and GA finished fabrics, but lower than that of BTCA-finished fabrics (Fig. 5a). This was an indication that the carboxyl and aldehyde groups of openSu cross-linked with the cotton fabric and imparted the anti-wrinkle properties through the two-step curing process^4,5,11^. From The WI of openSu treated fabric was 72.1, which was higher than those of GA (65.5) and BTCA (61.4) (Fig. 5b). This could be attributed to the dehydration and condensation of the finishing agent or the hydroxyl group of cellulose during the curing process to form unsaturated and colored compounds, which affected the whiteness of the fabric^3,8,19^.

The openSu-finished fabric had the shortest wetting time (less than 3 s), which was similar to the reference but much shorter than the wetting time of DMDHEU-, BTCA- and GA-treated fabrics (more than 42 s). This suggests that the higher the anti-wrinkle performance of the finished fabrics, the poorer the hydrophilicity performance as seen with the BTCA, DMDHEU and GA finished fabrics. The short wetting time of openSu could be attributed to its molecular structure which contains reactive groups-aldehyde groups, carboxyl groups and hydrophilic groups-hydroxyl groups. The aldehyde and carboxyl groups were used as cross-linking groups to react with the hydroxyl groups of cellulose. During the reaction process, the hydroxyl groups of the cellulose were consumed, and at the same time, the hydroxyl groups of openSu were introduced into the cellulose. As a result, there was little change in the hydroxyl group content of the finished fabric, and the wetting time of the fabric was shorter than the other finishing agents. Therefore, openSu has obvious advantages in improving the WRA, WI, TS and hydrophilicity properties of finished fabric.

**Figure 6:**
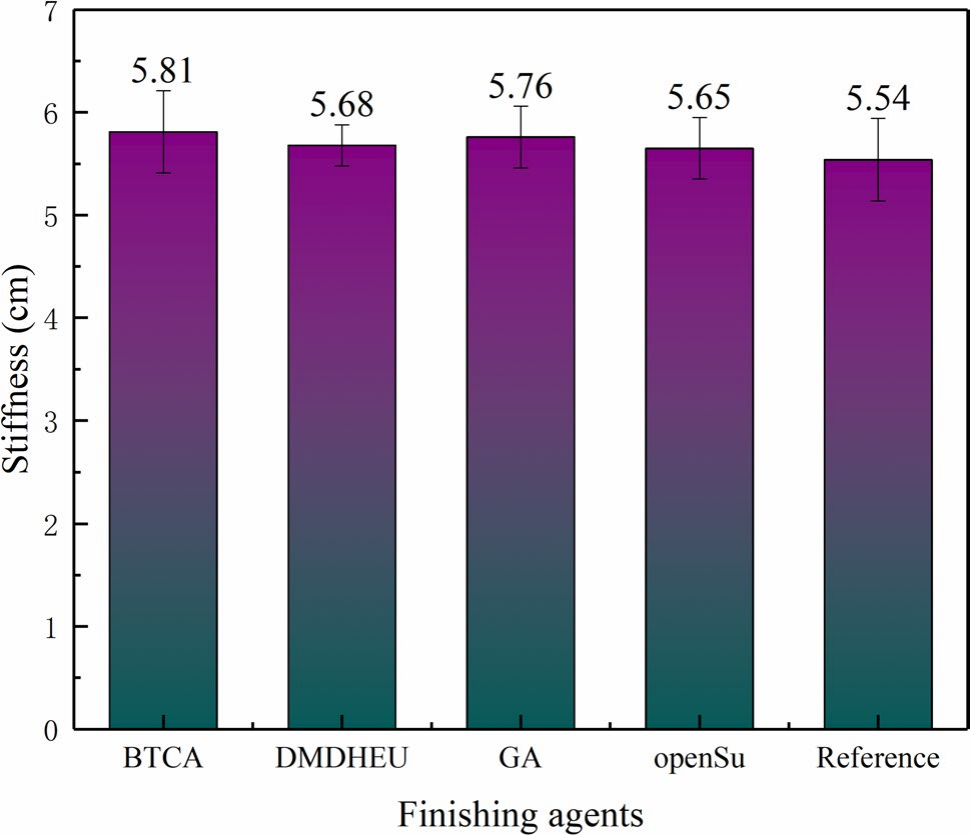
The stiffness of the finished fabrics with different finishing agents.

Fabric stiffness was one of the fabric hand feeling values, which represented the ability of textile materials to resist bending deformation. We compared the stiffness of the fabrics finished with different finishing agents (Fig. 6). In Fig. 6, the stiffness of openSu finished fabric was 5.65 cm, it is 1 cm longer than the reference, which was similar to that of the DMDHEU finished fabric. The stiffness of BTCA and GA finished fabric was 5.81 cm and 5.76 cm, similarly, the WRA of their finished fabrics was also higher. Combined with the WRA of their finished fabric, it can be inferred that the stiffness of the fabric was related to WRA, and the fabric with higher WRA had greater stiffness.

### Crosslinking mechanism between openSu and cellulose

**Figure 7:**
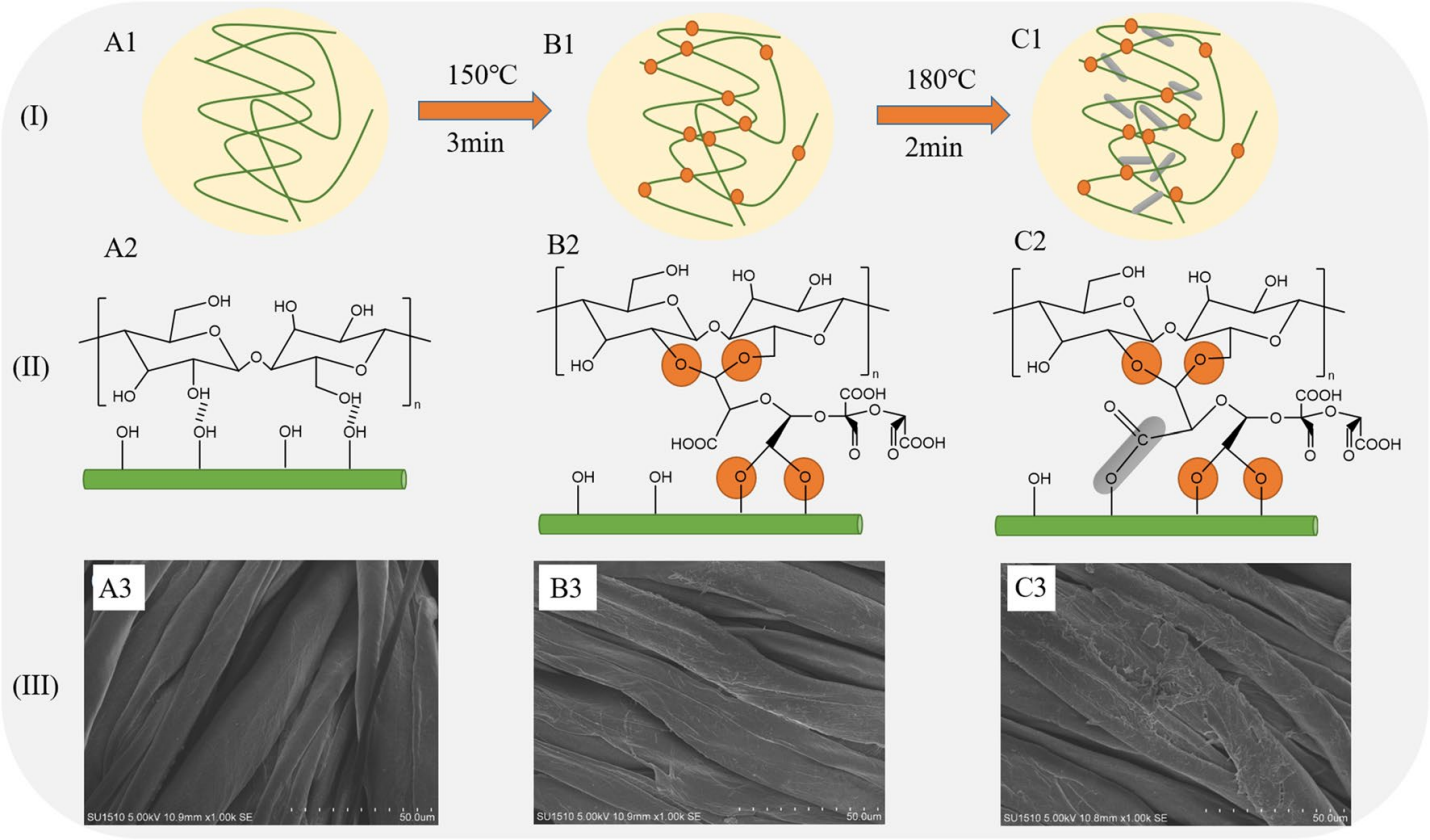
Possible reaction mechanism (I, II), SEM images (III) of reference (A1, A2, A3), openSu (150 °C) (B1, B2, B3) and openSu (150 °C and 180 °C) (C1, C2, C3).

Based on the results of ^13^C NMR and anti-wrinkle finishing analysis, we proposed a possible crosslinking mechanism between openSu and cotton cellulose (Fig. 7). The SEM images of fabrics finished with oxySu and openSu are also shown in Fig. 7.

Figure 7I shows a scheme representing the combination of openSu and cotton fabric under different curing temperatures. From the scheme we see that there was some cross-linking between openSu and cellulose after curing at 150 °C for 3 min, and full cross-linking was achieved after curing at 180 °C for 2 min.

Figure 7II shows a scheme representing the combination between the aldehyde groups and carboxyl groups of openSu and hydroxyl groups of the fiber after the two curing processes. The aldehyde groups reacted with the primary hydroxyl groups in the fiber during the curing process at 150 °C, to confer some anti-wrinkle properties to the fabric^4,8,9,22^. At 180 °C, the carboxyl groups of openSu combined with cellulose^5,11^, to further improve the WRA of the finished fabric.

Figure 7III shows the SEM images of the cotton fabric treated using openSu in the two-step curing process. From the images, we see that the surface of the openSu treated fabric has obvious attachments, and that the surface of cellulose was rough. In addition, the number of attachments was higher in the openSu treated fabrics cured at 150 °C and 180 °C than in the openSu fabrics cured at only 150 °C. These findings indicated that there were two-step cross-linking reactions between the aldehyde and carboxyl groups with cellulose when openSu was cured at 150 °C and 180 °C. These reaction are the etherification addition reaction and esterification reaction^6,15,21^.

## Conclusions

In this paper, openSu was successfully prepared through selective oxidation by the TEMPO-laccase and NaIO_4_ system. Results of ^13^C NMR analysis showed that openSu had multiple aldehyde and carboxyl groups, which was a reflection of its high reactivity. This article focused on the effects of the catalytic system, and curing conditions on the openSu-finished fabric. The optimal catalytic system was a mixed catalyst of MgCl_2_ and SHP, at a ratio of 16 g/L:4 g/L. The optimal curing conditions were 150 °C for 3 min followed by 180 °C for 2 min. Under these conditions, the aldehyde and carboxyl groups of openSu were fully cross-linked with cellulose, and greatly improved the anti-wrinkle performance of the finished fabric. The performance of the openSu-finished fabric was similar to that of DMDHEU and GA treated fabrics, but much lower than that of BTCA treated fabric. The hydrophilicity of the openSu-finished fabrics was higher than the other treated fabrics, but similar to that of the reference sample. Therefore, openSu has significant advantages in the anti-wrinkle finishing of cotton fabrics.

## Experimental section

### Material and methods

Scoured and bleached cotton woven fabric (133 warp yarns and 100 wefts per 10 cm, weighs 133 g/m^2^) was purchased from Shandong Lutai Group Co., Ltd. Zibo, China. Sucrose (Su), hydroxyl amine hydrochloride, magnesium chloride (MgCl_2_), sodium hypophosphite (SHP), NaIO_4_, and GA were purchased from Sinopharm Chemical Reagent Co., Ltd. Shanghai, China. TEMPO, Laccase (0.5U/mg), DMDHEU and BTCA were purchased from J&K Scientific Ltd. Beijing, China. All chemical reagents were of reagent grade.

### Two-steps selectively oxidation of openSu

1. TEMPO-mediated oxidation of sucrose was carried out as previously described with slight modifications^13,17,24^. Briefly sucrose (1.026 g) was mixed with acetate buffer solution (200 mL, pH was adjusted to 5.0) in a thermostatic magnetic water bath using a stirrer for 2–3 h. Afterwards, 95% ethanol was used to stop the reaction and then the pH was adjusted to 7.0 using 0.5 mol/L HCl. A membrane separation system was used to remove TEMPO and laccase to obtain the 6, 6′-carboxy sucrose (oxySu) solution. The oxySu solution was freeze-dried to obtain oxySu powder.
2. Selective oxidation of oxySu using NaIO_4_ was carried out as previously described with slight modifications^7,15,16,21^. OxySu (6.84 g) was mixed with deionized water (200 mL), stirred and bowled in nitrogen (N_2_) for 0.5 h to remove O_2_. The temperature of the solution was then adjusted to 10–15 °C, NaIO4 added (12.80 g) and the mixture stirred for 20–24 h. After the reaction, BaCl_2_ (14.67 g) was added and the mixture stirred for an additional 30 min. The precipitate was filtered to obtain the openSu solution, which was freeze-dried to obtain the openSu powder.

### Effect of TEMPO-laccase reaction conditions

The “single factor selection method” was used to determine the effect of oxidation reaction conditions on the TEMPO-laccase oxidation process. Four parameters including the amount of TEMPO and Laccase as well as the temperature and reaction time were studied. The optimal values for each parameter were determined and used in subsequent experiments.

### Carboxyl group content

The carboxylate content of openSu was determined through titration. OpenSu powder (0.25 g) was dissolved in 50 mL deionized water, and the pH of the solution adjusted to 2.5 using 0.1066 mol/L hydrochloric acid standard solution. The solution was then titrated by adding 0.50 mL of 0.05 mol/L sodium hydroxide standard solution dropwise until an equilibrium was achieved. The pH value at this point was recorded. The volume of sodium hydroxide solution consumed during titration was taken as the abscissa, while the pH value was taken as the ordinate to draw a graph to obtain a curve with two break points. The first break point corresponded to the volume of the sodium hydroxide solution V_1_ (mL), and the second break point was V_2_ (mL). The total carboxyl group content A (mmol/g) was calculated using the following formula:

1 $${\text{A}} = \left( {V_{2} - V_{1} } \right) \times n_{NaOH} /W$$

where n_NaOH_ was the concentration of NaOH (mol/L), and W was the weight of openSu.

### Aldehyde group content

Hydroxylamine hydrochloride-point titration was used to measure the aldehyde group content of openSu. OpenSu (0.100 g) powder was mixed with 25 mL of 0.25 mol/L hydroxylamine hydrochloride solution, followed by the addition of 2 drops of 0.05% methyl orange solution. The mixture was left to stand for 2 h to allow all the components to fully dissolve. The mixture was then titrated using 0.1 mol/L NaOH solution. The titration was stopped when the solution turned from red to yellow (pH about 4), and the volume ΔV of NaOH consumed recorded. The aldehyde content of openSu (mmol C = OH/g openSu) was calculated using Eq. (2):

2 $${\text{B}} = \varDelta V \times 0.001 \times n_{NaOH} /w$$

where ΔV was the volume of NaOH (mL) consumed, n_NaOH_ was the concentration of NaOH (mol/L), *w* was the weight of openSu.

### ^13^C NMR analysis

Sucrose (20 mg), oxySu and openSu were dissolved in 550 μL deuterium water (D_2_O), and then nuclear magnetic resonance (^13^C-NMR) detection carried out using a Bruker AduanceIII 400 MHz nuclear magnetic resonance spectrometer. The internal standard was tetramethylsilane (TMS).

### Anti-wrinkle finishing of cotton fabric

The finishing solution was prepared by mixing a certain concentration of openSu anti-wrinkle finishing solution with penetrant JFC-2 10 g/L and polyethylene softener 10 g/L. Different mass concentration ratios of the catalyst MgCl_2_ and NaH_2_PO_2_ were used to give a total mass concentration of 20 g/L.

The finishing process as follow: padding finishing solution (two dipping and two rolling, dipping temperature 25 °C, wet pick-up was 85–90%) using a two-bowl horizontal laboratory padder (Yalinuo, Guangzhou, China), and drying at 80 °C for 3 min), then curing at different curing conditions.

### WRA analysis

The WRA of the treated fabric was determined according to the AATCC Test Method 66-2003 “Textile fabric crease recovery measurement method of recovery angle”. Using 5 warps and 5 wefts, and the data was presented as the average of three sets of data.

### WI analysis

The WI of the finished fabric was determined according to AATCC Test Method 110-2005 “Textile Color Fastness Test Relative WI Instrument Evaluation Method” using a Datacolor 650^®^ Bench top spectrophotometer. The average value was taken after 5 measurements.

### TS analysis

The strength of the fabric was determined according to ASTM Testing Method D-1424-1996 “Testing Method for the Breaking Strength and Breaking Elongation of Woven Fabrics”, using the HD026NS electronic fabric strength tester. The fabric was cut into a length of 250 mm ± 0.5 mm and a width of 50 mm ± 0.5 mm. TS was calculated according to the following formula (%):

3 $$TS = T_{t} /T_{u} *{1}00\%$$

where *T*_t_ was the tensile strength (cN) of finished fabric and *T*_u_ was the tensile strength (cN) of unfinished fabric.

### Hydrophilicity analysis

The hydrophilicity of the finished product was determined according to AATCC 79-1995. A drop of water was dropped on 5 randomly selected points of the fabric, and the time taken for the droplet to disappear recorded. The average value for the 5 points was calculated and was considered to be the wetting time of the fabric.

### SEM analysis

The finished fabric was dried and then sprayed with gold. SEM analysis was carried out using the SU1510 scanning electron microscope (Hitachi, Suzhou, China). The acceleration voltage was set at 5 kV, and the fiber surface morphology was observed at a magnification of 1000 times.

### Stiffness analysis

According to standards of test methods “ISO 9073-7-1995 Textiles—Test methods for nonwovens—Part 7: Determination of bending length”, the stiffness of the finished fabrics with different finishing agents were measured.

## References

[1] Dehabadi VA, Buschmann H-J, Gutmann JS (2013). Durable press finishing of cotton fabrics: An overview. Text. Res. J..

[2] Harifi T, Montazer M (2012). Past, present and future prospects of cotton cross-linking: New insight into nano particles. Carbohydr. Polym..

[3] Ji B, Tang P, Yan K, Sun G (2015). Catalytic actions of alkaline salts in reactions between 1,2,3,4-butanetetracarboxylic acid and cellulose: II. Esterification. Carbohydr. Polym..

[4] Chang H-L, Chen C-C (2016). Crosslinking of cotton with DMDMDHEU in the presence of sodium chloride. Text. Res. J..

[5] Zhang X, Ji B, Yan K, Hu T (2017). Non-phosphorus catalysts for the ester cross-linking of cellulose with 1,2,3,4-butanetetracarboxylic acid. Fiber Polym..

[6] Liu P, Xu H, Mi X, Xu L, Yang Y (2015). Oxidized sucrose: A potent and biocompatible crosslinker for three-dimensional fibrous protein scaffolds. Macromol. Mater. Eng..

[7] He X, Tao R, Zhou T, Wang C, Xie K (2014). Structure and properties of cotton fabrics finished with functionalized dialdehyde chitosan. Carbohydr. Polym..

[8] Fu Y, Michopoulos J, Song JH (2015). Coarse-grained molecular dynamics simulations of epoxy resin during the curing process. Comp. Mater. Sci..

[9] Chen JC (2001). Crosslinking of cotton cellulose with pre-reacted DMDMDHEU-AA part II: Reaction kinetics. Text. Res. J..

[10] Chen JC, Chen CC (2001). Crosslinking of cotton cellulose with pre-reacted DMDMDHEU-AA. Part I: Physical properties of finished fabrics. Text. Res. J..

[11] Wang H, Zhang C, Chu X, Zhu P (2020). Mechanism of antiwrinkle finishing of cotton fabrics using mixed polycarboxylic acids. Int. J. Polym. Sci..

[12] Hoover JM, Ryland BL, Stahl SS (2013). Copper/TEMPO-catalyzed aerobic alcohol oxidation: Mechanistic assessment of different catalyst systems. ACS Catal..

[13] Iron MA, Szpilman AM (2017). Mechanism of the copper/TEMPO-catalyzed aerobic oxidation of alcohols. Chemistry.

[14] Mendoza DJ, Browne C, Raghuwanshi VS, Simon GP, Garnier G (2019). One-shot TEMPO-periodate oxidation of native cellulose. Carbohydr. Polym..

[15] Wang P (2018). Synthesis and characterization of corn starch crosslinked with oxidized sucrose. Starch.

[16] Muhammad M, Willems C, Rodriguez-Fernandez J, Gallego-Ferrer G, Groth T (2020). Synthesis and characterization of oxidized polysaccharides for in situ forming hydrogels. Biomolecules.

[17] Liu H, Liu X, Yue L, Jiang Q, Xia W (2016). Synthesis, characterization and bioactivities of N, O-carbonylated chitosan. Int. J. Biol. Macromol..

[18] Pourjavadi A, Aghajani V, Ghasemzadeh H (2008). Synthesis, characterization and swelling behavior of chitosan–sucrose as a novel full-polysaccharide superabsorbent hydrogel. J. Appl. Polym. Sci..

[19] Badalyan A, Stahl SS (2016). Cooperative electrocatalytic alcohol oxidation with electron–proton–transfer mediators. Nature.

[20] Lou J, Yuan J, Wang Q, Xu J, Fan X (2020). Anti-crease finishing of cotton fabrics based on crosslinking of cellulose with oxidized sucrose. J. Nat. Fibers.

[21] Lou J (2019). Oxysucrose polyaldehyde: A new hydrophilic crosslinking reagent for anti-crease finishing of cotton fabrics. Carbohydr. Res..

[22] Munster L (2018). Dialdehyde cellulose crosslinked poly(vinyl alcohol) hydrogels: Influence of catalyst and crosslinker shelf life. Carbohydr. Polym..

[23] Bu X (2019). Effect of carboxyl content on the physicochemical properties and intramolecular water distribution of 6-carboxyl chitooligomer prepared by laccase-TEMPO. J. Appl. Polym. Sci..

[24] Liang X, Trentle M, Kozlovskaya V, Kharlampieva E, Bonizzoni M (2019). Carbohydrate sensing using water-soluble poly(methacrylic acid)-*co*-3-(acrylamido)phenylboronic acid copolymer. ACS Appl. Polym. Mater..

